# Isolated Right Temporal Lobe Stroke Patients Present with Geschwind Gastaut Syndrome, Frontal Network Syndrome and Delusional Misidentification Syndromes

**DOI:** 10.3233/BEN-2008-0218

**Published:** 2009-07-29

**Authors:** Michael Hoffmann

**Affiliations:** Division of Cognitive NeurologyDepartment of NeurologyUniversity of South Florida and VA James A Haley HospitalTampaFL 33612USA

## Abstract

*Background:* Right temporal lobe lesion syndrome elicitation presents a clinical challenge. Aside from occasional covert quadrantanopias, heralding elementary neurological deficits are absent.

*Aim:* Isolated right and left temporal lobe stroke patients were analyzed for the panoply of known temporal and frontal cognitive and neuropsychiatric syndromes.

*Methods:* Temporal lobe stroke patients were analyzed, derived from a dedicated cognitive stroke registry. Patients were screened by a validated bedside cognitive battery and a neuropsychological test battery, including the Bear Fedio Inventory for diagnosis of the Geschwind Gastaut (GG) syndrome, frontal network syndrome testing (FNS), emotional intelligence testing and delusional misidentification syndromes (DMIS). NIH stroke scores were documented and lesion location identified with the 3 dimensional digitized Cerefy coxial brain atlas. Exclusions were coma, encephalopathy and medication related effects.

*Results:* Of 2389 patients analyzed, in patients with isolated right temporal lobe (IRT) stroke (*n* = 5, infarcts *n* = 3, hemorrhage *n* = 2), the GG syndrome and FNS were present in all five. Other relatively frequent syndromes included DMIS in 4, visuospatial dysfunction in 2 and amusia in 2. No patient had a NIHSS greater than 1. The only elementary neurological sign was quadrantanopia in 3 patients. Lesion location was mid and lateral temporal lobe (*n* = 2), middle and mesial temporal lobe (*n* = 1) middle temporal lobe (*n* = 1) and lateral temporal lobe (*n* = 1). Comparison with isolated left temporal lobe (ILT) stroke revealed syndromes of aphasia (*n* = 4), alexia (*n* = 2), acalculia (*n* = 2), agnosia (*n* = 2), verbal amnesia (*n* = 1), none of which occurred in the IRT patients. The mean NIHSS scores of IRT (0.6) and ILT strokes (4.2) was different (*t* = 2.23, *p* = 0.04). The 2 × 8 Fisher Exact Test revealed significant differences for the clusters of syndromes occurring in the right and left isolated temporal lobe lesions (*p* = 0.00002).

*Conclusion:* The GG syndrome, FNS and DMIS are prominent syndrome constellations in stroke patients involving the right temporal lobe and constitute the neurological deficit without heralding long tract signs. By extrapolation these syndromes may also be present in the general right hemisphere lesion population.

## 1. Background

The neurological literature still refers to silent brain lesions, most notably those affecting the frontal lobes, temporal lobes and parts of the parietal lobes, areas coinciding with tertiary association cortex. Whereas lesions affecting most parts of the left hemisphere invariably present with differing aphasia syndromes, right temporal lobe syndrome elicitation in particular presents a clinical challenge. Aside from occasional covert quadrantanopias, heralding elementary neurological deficits are absent. Crude measures such as the Minimental State Examination (MMSE) and NIH stroke score (NIHSS) are insensitive to tertiary association cortex. Nevertheless elicitation of a brain lesion whether silent or not represents an opportunity to mea-

ISSN 0953-4180/08/$17.00 © 2008 – IOS Press and the authors. All rights reserved sure deficit, monitor progress, intervene and prevent further injury.

## 2. Aim

Isolated right and left temporal lobe stroke patients were analyzed for known temporal and frontal cognitive syndromes.

## 3. Methods

Consecutive stroke patients, aged 18-90 years were accrued through a prospectively coded, dedicated cognitive stroke registry, as part of a tertiary care Joint Commission on Accreditation of Healthcare Organizations (JCAHO) primary and Agency for Health Care Administration (AHCA) Comprehensive Stroke Center (Florida). All patients were examined and managed by board certified neurologists. The Stroke registry was approved by the University Institutional Review Board and in compliance with HIPAA regulations. All patients signed informed consent for the evaluation and the collection of their neurological, medical and neurocognitive data.

### *3.1. Clinical parameters and cognitive metric testing*

All stroke patients were tested with a validated bedside cognitive battery [1] within one month of stroke onset. This allowed screening for a large number of behavioral neurological and neuropsychiatric syndromes. If positive for any cognitive syndromes, specific neuropsychological tests followed. Patients with Isolated right and left temporal stroke were analyzed further. These included frontal tests, better termed frontal network syndrome (FNS) tests, that measured executive functioning, apathy and disinhibition by the Frontal Systems Behavioral Scale (FRSBE) [2], emotional intelligence (EI), (Baron EQ test) [3] and the Wisconsin Card Sorting Test (WCST) for executive functioning [4]. The Bear Fedio Inventory was used for detection of the Geschwind Gastaut (GG) syndrome [5] and delusional misidentification syndromes (DMIS) diagnosed by DSM IV criteria [6] and subtypes according to Feinberg et al. [7]. Patient follow up extended up to 5 years post event.

### *3.2. Neuroimaging*

Multimodality MRI (T1, T2), fluid attenuation inversion recovery (FLAIR), diffusion weighted imaging (DWI) magnetic resonance angiography (MRA) (intracranial and cervicocephalic) was performed in all patients when feasible and no contra indication to magnetic resonance imaging existed. Board certified neuroradiologists associated with the Comprehensive Stroke Center evaluated the MRI images and had no information regarding the cognitive syndromes. Temporal lobe localization and stroke lesion location was determined with the aid of the 3 dimensional digitized Cerefy coxial brain atlas [8]. NIH stroke scores were tabled to ascertain overall neurological vascular deficit.

### *3.3. Geschwind Gastaut syndrome diagnosis with the bear fedio inventory*

The three principal features that characterize this syndrome are i) a viscous personality, ii) metaphysical preoccupation and iii) altered physiological drives. The viscous personality is considered the central component of the GG syndrome and includes circumstantiality in speech, over inclusive verbal discourse with excessive detail of information, stickiness of thought processes, interpersonal adhesiveness, prolongation of interpersonal encounters and hypergraphia that can take the form of excessive writing, drawing or painting [5]. The metaphysical preoccupation component may take the form of nascent and excessive intellectual interests in religion, philosophy or moral issues. The third principal component includes alteration of physiological drives such as alteration in sexuality, mostly hyposexuality, aggression and fear. For the purposes of diagnosis, the requirement was two of the three principal features plus one or more of the other Bear Fedio Inventory features.

### *3.4. Delusional Misidentification Syndromes (DMIS) definitions*

A person is considered to have a content specific delusion when he/she incorrectly identifies or reduplicates persons, places, objects or events [6].

This information may be gleaned by self-report or by family members or friends. All stroke patients were also directly questioned regarding delusions of any form by the examiner. If present, the delusion description was compared to one of the four commonly recognized entities but at least 15 different rarer varieties have been reported [6,7,9–11]. These included the Capgras syndrome where the patient believes that a familiar individual or the patient him/herself has been replaced or substituted by a double or imposter (hypoidentification syndrome), Fregoli's syndrome where a person who is familiar to the patient is really impersonating and thus takes on the appearance of a stranger (hyperidentification syndrome), Intermetamorphosis where two individuals known to the person have exchanged identities with each other and reduplicative paramnesia where the patient reports geographical location or place duplication.

### 3.5. *Statistical evaluation*

Descriptive analysis, including means, 95% confidence intervals and standard deviations for continuous variables and frequency distributions for categorical variables, were obtained for all study parameters. T-tests were used to compare means and a p value of $\leqslant$ 0.05 was regarded as significant. Categorical data was evaluated with the Chi Square Test and for cell sizes less than five Fisher's Exact Test was used. Associations between continuous variables were analyzed using Pearson's product moment correlation. All analyses were run in SAS version.

## 4. Results

The patient cohort ($n$ = 2389) demographic details included a mean age of 62.4 years (SD = 16.38), gender – female 1187 (49.7%), race ethnicity included white 1717 (71.9%), African American 352 (14.7%), Hispanic 157 (6.6%) and other 163 (6.8%). Handedness included right 1664 (69.7%), left 45 (1.9%), ambidextrous 12 (0.5%) and uncertain in 668 (27.9%). Coma ($n$ = 216) and encephalopathy ($n$ = 144) were excluded from the analyses for cognitive evaluation in the registry except for the 11 who recovered from coma and the 65 (total subtracted from 2389–284 = 2105) who recovered from encephalopathy sufficiently within the first month to allow cognitive testing. The 309 TIA patients were not included in the remainder of the analysis yielding the study number of 1796.

Syndromes associated with the right hemisphere network included $n$ = 275/1796 (15.3%) instances with components of neglect ($n$ = 173), anosognosia ($n$ = 115), visuospatial dysfunction ($n$ = 70) and aprosodia ($n$ = 43) and involuntary emotional expression disorder (IEED) of which 10/12 (83%) instances occurred

Table 1
Isolated right temporal lobe stroke, Cognitive Metric Test Batteries, DMIS and GG instances and NIHSS

| | FRSB | WCST | EQ | DS | GGS | NIHSS |
|---|---|---|---|---|---|---|
| Patient # | | | | | | |
| 1 | A 100*<br>E 87* | 50 | 75* | 1 | 3 | 1 |
| 2 | A 63*<br>D 63* | 35* | 80* | 2 | 3 | 1 |
| 3 | D 69* | 57 | 118 | 1 | 2 | 0 |
| 4 | A 101*<br>E | 31*<br>73* | 100 | 1 | 2 | 1 |
| 5 | A 83*<br>D 62*<br>E 77* | 25* | 65* | 2 | 3 | 0 |

FRSB: Frontal Systems Behavioral Inventory.
A: Apathy.
D: Disinhibition.
E: Executive function.
WCST: Wisconsin Card Sorting Test.
EQ: Emotional intelligence quotient.
DS: Delusional Misidentification Syndrome (number of different types present).
GGS: Geschwind Gastaut Syndrome (number of the 3 components present).
NIHS: National Institutes of Health Stroke Score.
*Denotes abnormal result in T scores (FRSB, WCST) or standard scores(EQ).

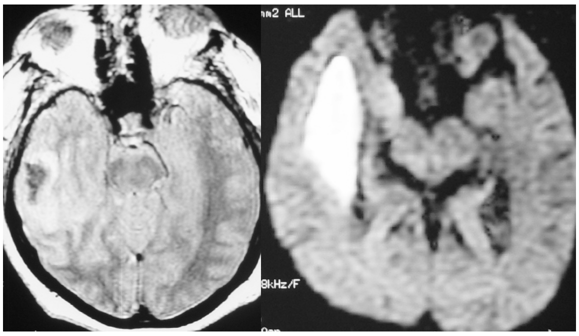

Fig. 1. Isolated right temporal lobe hemorrhagic infarct (A) from patient 4 and hemorrhage (B) from patient 2.

with right hemisphere lesions. Those lesions, isolated to the right temporal lobe ($n$ = 5, 0.2%, infarct $n$ = 3 and hemorrhage $n$ = 2) were analyzed further. A representative isolated right temporal lobe hemorrhage and hemorrhagic infarct is noted in Fig. 1. Demographics included 3 men, 2 women with mean age 57.2 years, range 50–66 years. Exclusions included extratemporal involvement by the lesion, coma, cerebral trauma, encephalopathy, substance abuse, neurodegenerative disease and medication related effects. All 5 patients had the Geschwind Gastaut syndrome and 3/5 patients had all 3 principal components present of the GG syndrome. All five patients also had one or more frontal network

Table 2
Comparison of the clustering of cognitive domains of right and left isolated temporal lobe stroke

| | Cognitive domain | | | | | | | | |
|---|---|---|---|---|---|---|---|---|---|
| | GGS | DS | FNS | A | Al | Ag | Ac | VS | Am |
| RT | 5 | 5 | 5 | 0 | 0 | 0 | 0 | 2 | 2 |
| LT | 0 | 0 | 1 | 4 | 2 | 2 | 2 | 0 | 0 |

Fisher's Exact Test 2x8 table: $p = 0.00002$.
GGS: Geschwind Gastaut Syndrome.
DS: Delusion Misidentification Syndrome.
FNS: Frontal Network Syndrome.
A: Aphasia.
Al: Alexia.
Ag: Agnosia.
Ac: Acalculia.
Am: Amusia.
VS: Visuospatial dysfunction.
RT: Right temporal lobe.
LT: Left temporal lobe syndromes (FNS) either apathy, disinhibition or dysexecutive impairment singly, or in combination. These included apathy in 4/5, dysexecutive functioning in 3/5, disinhibition in 3/5, abnormal WCST error percentage T scores in 3/5 and abnormally low EI quotient scores (less than 90 in standard scores) in 3/5. Four of 5 patients had delusional misidentification syndromes and two patients had more than one DMIS (Table 1). Less frequent syndromes included visuospatial dysfunction ($n = 2$) and amusia ($n = 2$). Elementary neurological deficits included quadrantanopias ($n = 3$) and NIHSS were minimal, scoring 1 in 3 patients and 0 in 2.

Comparison was made to isolated left temporal lobe lesions in this registry and notably no patient had GG or DMIS with one patient manifesting with FNS. The syndromes here included an aphasic syndrome $n = 4$, alexia $n = 2$, acalculia $n = 2$, agnosia $n = 2$, verbal amnesia $n = 1$, none of which occurred in the IRT patients (Table 2). The mean age of the IRT versus the ILT patients was 54.4 and 70.6 years without a gender difference (2 women in each group). The mean NIHSS scores of right temporal lobe (0.6) and left temporal lobe (4.2) was different ($t = 2{,}23$, $p = 0.04$). The $2 \times 8$ Fisher Exact Test revealed significant difference for the clusters of syndromes occurring in the right and left isolated temporal lobe lesions ($p = 0.00002$). The individual patient clinical summaries are noted in appendix 1 and representative infarct (patient 4) and hemorrhage (patient 2) lesions depicted in Fig. 1.

## 5. Discussion

Five main pathologies commonly affect the temporal lobe; epilepsy, stroke, encephalitis, tumors, trauma. The only recognized syndrome complexes include the Kluver Bucy syndrome (KBS) and GGS although other individual symptoms may manifest as well, including amnestic syndromes and neuropsychiatric entities such as anxiety, depression, rage attacks, and mania [11–16]. The KBS is exceedingly rare and occurs only with bilateral lesions and the GGS has only been described in association with epilepsy [14,15,17]. As noted in this case series, although isolated stroke lesions of the right temporal lobe are also relatively rare, the temporal lobe is very often involved in larger vascular lesions within the middle cerebral artery territory, the most frequently involved artery in stroke. The right temporal lobe has for a long time been regarded as a "clinically silent area" of the brain by clinicians and neurosurgeons. However, the GG syndrome, FNS and DMIS are prominent syndrome constellations in stroke patients involving the right temporal lobe and constitute the neurological deficit, importantly, often without heralding long tract signs. These cognitive syndromes as described may also be present in the context of other middle cerebral artery syndromes. The constellation of isolated temporal cognitive syndromes differs markedly between the two hemispheres. Left temporal lobe lesions are dominated by dysphasic syndromes together with other language related deficits such as alexia, acalculia but also occasional agnosias and dyschromatopsia. Right hemisphere temporal lesions were dominated by FNS, DMIS and GGS in all patients with 2 cases of visuospatial dysfunction and one with amusia. One person with ILT stroke had a mild frontal network syndrome, but it is possible that language barriers precluded comprehensive FNS assessment in at least 2/5 ILT patients. It is acknowledged that the presence of

Table 3
Proposed temporal lobe cognitive and neurospsychiatric presentations, modified from Devinsky O [13]

| |
|---|
| **Left** |
| Elementary: |
| Right upper quadrantanopia |
| Vestibular: vertigo and dysequilibrium |
| Neuropsychiatric: |
| Delirium, paranoia, agitation, aggression, depression |
| Cognitive: |
| Language: Aphasia: Wernicke's, transcortical sensory and anomic |
| Memory: Verbal amnesia |
| Visual agnosia |
| Amusia |
| Disorders of time perception |
| Synesthesia |
| **Right** |
| Elementary: |
| Left upper quadrantanopia |
| Vestibular: vertigo and dysequilibrium |
| Neuropsychiatric: |
| Delirium, paranoia, agitation, psychosis |
| Cognitive: |
| Delusional Misidentification Syndrome |
| Geschwind Gastaut Syndrome |
| Memory: Visuospatial amnesia |
| Auditory agnosia |
| Receptive amusia |
| Disorders of time perception |
| **Right or left** |
| Frontal Network Syndrome |
| **Bilateral** |
| Kluver Bucy Syndrome (or individual components thereof such as placidity and agnosia) |
| Neuropsychiatric: |
| Delirium, paranoia, aggression, agitation |
| Cognitive: |
| Memory: Korsakoff amnestic state |
| Cortical deafness |
| Auditory agnosia |
| Prosopagnosia (boundary between occipital and temporal lobes) |

dysphasia of varying degrees and subtypes, may render the cognitive testing approach incomplete. One patient with Wernicke's aphasia with poor comprehension and one patient with global aphasia were not further testable.

To my knowledge no reports of GGS in association with stroke have been published at the time of writing. The available literature concerning GGS is almost entirely in association with temporal lobe pathology in the context of epilepsy. Content specific delusions have most often been noted with right hemisphere lesions and more specifically with right temporal lobe lesions [9]. The associated pathologies have been tumor and epilepsy, with those due to stroke the minority of reported cases [9,10].

Although isolated temporal lobe lesions in cerebrovascular disease are relatively rare, caused mainly by intracerebral hemorrhage and inferior division of the middle cerebral artery infarction, associated syndrome elicitation is nevertheless important. Standard stroke scales, bedside cognitive tests and neuropsychological tests are insensitive to their presence. In addition, many stroke syndromes and other neurological conditions involve the temporal lobes as part of a more extensive lesion and the above cognitive syndrome presentation is relevant to such patients to tease out more precisely the nature of their deficits.

The small number of patients does not allow any further deductions to be made about other well known temporal lobe presentations such as verbal and visuospatial memory disorders nor depression or anxiety which were not diagnosed clinically by the DSM IV criteria [6]. It has been aptly stated that the temporolimbic area is in the borderland between psychiatry and neurology [18]. It behooves us therefore to maintain a cross disciplinary view of the presentation of symptoms, signs and syndromes in this brain circuitry. In an attempt to foster clarity of the behavioral neurological and neuropsychiatric presentations, a modification of previous suggestions is presented in Table 3. It is recommended that as clinicians we should get into the habit of describing brain illness presentations with respect to 1. elementary neurological deficits (for example; weakness, sensory loss, visual field loss, imbalance), 2. cognitive neurological (for example; frontal network syndromes, aphasias, neglect syndromes, aprosodia) and 3. neuropsychiatric (for example; depression, anxiety, mania, involuntary emotional expression disorder, GGS). For neurologists and psychiatrists working in the borderland areas, we will have to improve our understanding and learn to speak the language of both disciplines.

**Acknowledgements**

Presented at the American Neuropsychiatric Association annual meeting in San Diego, February 2006.

Dr. Ren Chen MD, Biostatics Core of the University of South Florida, Tampa, Florida for advice and assistance with the statistical analyses.

## Appendix 1. Individual patient clinical summaries

*Patient 1. White man, right handed, aged 57 years*

He presented post motor vehicle accident with apparent black out and imaging revealed that he sustained a bland infarct affecting with right hemisphere, inferior division of the middle cerebral artery infarct without any cerebral trauma. On investigation the presumed etiology was an anticardiolipin IgM elevation. On examination he was orientated for 10/10 items, serial 7's 5/5 correct, speech normal for comprehension, fluency and repetition. Luria's 3 step motor sequence 3/5 correct and a word list generation test with the letter "S" he was able to generate >20 per minute but several repetitions. The Go no go paradigm was normal with 5/5 correct responses and proverb interpretation 5/5 correct. During the interview, inappropriate jocularity was noted as well as excessive discourse and elaborations of his condition (interpersonal viscosity). Reading and writing normal were normal as was object and color recognition normal. There was no aprosodia, however dysgraphia was profound. He reported an inability to recognize songs he has known for years and described music sounded like was coming through a bad speaker – "the melody was all wrong" (amusia). Visuospatial impairment was apparent with inability to draw a 3 dimensional object such as a cube. He has experienced mania since stroke, anger, paranoia and frequently delusional. He has had religious obsessions, shaved off all his hair, become particularly interested in Tauism. Elementary neurological deficits included only some sensory impairment of the left leg and specifically no left upper quadrantanopia.

*Patient 2, white man, right handed, aged 50 years*

He presented to the emergency room with headaches reportedly with a recent seizure and a history of alcohol excess, atrial fibrillation and smoking history. The MRI brain scan revealed a right medial temporal intracerebral hemorrhage with volume 18 cubic centimeters. On examination he had motor aprosodia, a word list generation test using the letter "S" resulted in 17 words per minute, the Go no go test was normal with 10/10 responses, Luria's motor sequence test 5/10,correct, the similarities and differences test was normal with 6/6 correct responses , the Rey Complex Figure was copied well but recall at 5 min extremely poor. He was noted to "rambles on and on when asked about his company and his work – with excessive, circumstantial information that seemed to have no bearing at all on the questions asked. He frequently reported that he was at his work location or domicile during his hospitalization and mistook the nursing staff for family members. Elementary neurological signs revealed a left upper quadrantanopia.

*Patient 3, white woman, right handed aged 65 years*

She presented with a syncopal episode thought to be a seizure witnessed by husband and the MRI brain scan revealed a right hemorrhagic appearing, temporal lesion, approximately 2.5 cm diameter and because of a mass like appearance, a right craniotomy and biopsy was performed which revealed microinfarcts, reactive glosis and acute necrotizing vasculitis. The catheter angiogram had revealed a small area of vasculitis seen in right posterior parieto temporal region. Examination revealed an alert and cooperative woman, orientated for 8/10 items, serial 7's 4/5 correct, memory 3/5 words recalled at 5 minutes, visuospatial function was impaired for 3 dimensional copying of a cube and the Three Words Three Shapes Test score was 3/3 with prompting for one word and 2/3 correct for shapes. Reading and writing was normal, speech fluent and comprehension and repetition intact. She volunteered several other symptoms such as that "she is in someone's else's world, not her own". She used to be very independent and sure of herself and now is frightened and "her emotions are taking control, feels very insecure". Her short term memory is a big problem for her now, she is short tempered. "Has been into the spiritual side since her illness" She divorced – "he could not live with her", is very interested in spirituality. Reads and talks excessively. She is now a practitioner of Reiki – a Japanese religion. Elementary neurological deficits were notable only for a left upper quadrantic field defect.

*Patient 4. White woman, right handed, aged 56 years*

She presented with severe headache, vertigo and vomiting and subsequent seizure. Her husband noted that she is a little more anxious and "worries about everything is much more intense in discussions", is much more vocal about her opinions what is happening in the world. She often repeats herself and talks much more than previously. On one occasion she described herself in a state where "she felt herself leaving her body" (autoscopy). On examination she was orientated for 10/10 items, serial 7's 4/5 correct, memory for 5 words at 5 min 5/5 correct, the word list generation test with the letter "S" yielded 16 words per minute and the Luria motor sequence test resulted in 8/10 correct responses. Reading and writing were normal, object and color recognition normal. No elementary neurological syndromes were elicited such as senorimotor, visual field impairments or imbalance. The MRI brain scan revealed a right temporal hemorrhagic infarct and MR venogram revealed a right lateral sinus thrombosis. It was presumed that her hormone replacement therapy had resulted in a hypercoagulation syndrome with cerebral venous thrombosis. A comprehensive prothrombotic work up had otherwise bee negative.

*Patient 5. White man, right handed, aged 58 years.*

He presented with left sided weakness with a history of peripheral vascular disease, diabetes mellitus and the MRI brain scan revealed right cerebral artery distribution ischemic infarct of the temporal lobe only. On examination he was abulic, orientated for 10/10 items, serial 7's 3/5 correct, memory for 5 words at 5 min 2/5 correct, Luria's motor sequence test 10/10 correct, the Go no go test yielded 10/10 correct responses and the word list generation test using the letter "S" resulted in 11 words per minute. He was no longer able to play the piano because he has "forgotten his music" (amusia). He often cited that he was in a different location to his actual one (while in hospital). His wife volunteered that he is much more philosophical and religious. He enrolled in a Seminary for studying the bible. His interviews were notable for markedly overinclusive detail. He has become very anxious and chews his fingernails. There were no elementary neurological deficits.
